# Using Virus Sequencing to Determine Source of SARS-CoV-2 Transmission for Healthcare Worker

**DOI:** 10.3201/eid2610.202322

**Published:** 2020-10

**Authors:** Nasia Safdar, Gage K. Moreno, Katarina M. Braun, Thomas C. Friedrich, David H. O’Connor

**Affiliations:** University of Wisconsin School of Medicine and Public Health, Madison, Wisconsin, USA (N. Safdar);; William S. Middleton Memorial Veterans Hospital, Madison (N. Safdar);; University of Wisconsin–Madison, Madison (G.K. Moreno, K.M. Braun, T.C. Friedrich, D.H. O’Connor)

**Keywords:** 2019 novel coronavirus disease, coronavirus disease, COVID-19, severe acute respiratory syndrome coronavirus 2, SARS-CoV-2, viruses, respiratory infections, zoonoses, transmission, healthcare worker

## Abstract

Whether a healthcare worker’s severe acute respiratory syndrome coronavirus 2 (SARS-CoV-2) infection is community or hospital acquired affects prevention practices. We used virus sequencing to determine that infection of a healthcare worker who cared for 2 SARS-CoV-2–infected patients was probably community acquired. Appropriate personal protective equipment may have protected against hospital-acquired infection.

Healthcare workers (HCWs) are at the front lines of the coronavirus disease (COVID-19) pandemic; their interactions with patients and in the community put them at risk for infection with severe acute respiratory syndrome coronavirus 2 (SARS-CoV-2) (*1*,*2*). Concern about whether HCWs are adequately protected from exposure while caring for patients has been fueled by limited availability of recommended personal protective equipment (PPE), in particular N95 respirators. Determining an HCW’s source of SARS-CoV-2 infection—community versus healthcare system—is crucial for evaluating the effectiveness of hospital infection control and PPE practices. Although SARS-CoV-2 infections in HCWs are often presumed to be acquired during the course of patient care, few reports unambiguously identify the source of acquisition. Forensic genomics, using viral sequencing, may be a promising approach.

We report a case of SARS-CoV-2 infection of an HCW at the University of Wisconsin–Madison (Madison, WI, USA) who performed direct care for 2 non–critically ill patients with confirmed SARS-CoV-2 infections (patients 1 and 2). The University of Wisconsin–Madison Institutional Board deemed this study to be quality improvement rather than research and therefore exempt from review.

At the time of this investigation, community prevalence of SARS-CoV-2 in Dane County, Wisconsin, was relatively low (cumulative prevalence »0.06% as of April 17, 2020). During this time, precautions in place included universal masking for HCWs, universal face covering for hospital visitors, and masking of symptomatic patients when entering the healthcare system. Hospitalwide hand hygiene compliance rates were 93%–96%.

While caring for patients 1 and 2, the HCW in this study wore a barrier facemask made to ASTM International (https://www.astm.org) standards, a face shield, reusable gowns, and nonsterile gloves. Four days after providing care for these patients, the HCW began experiencing headache, fever, and sore throat. A nasopharyngeal swab sample was positive for SARS-CoV-2 viral RNA. To establish the possible source of infection, we interviewed the HCW’s family member, who had experienced a febrile illness 8 days before the HCW’s onset of symptoms but was not tested initially because of limited testing availability. A nasopharyngeal swab sample from the family member was also positive for SARS-CoV-2 (Figure 1).

**Figure 1:**
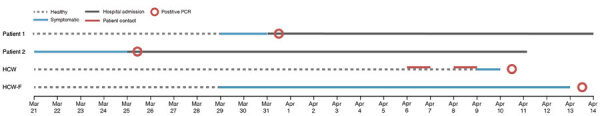
Timeline of infection, contact, and testing of HCW, HCW’s family member, and coronavirus disease patients 1 and 2, Madison, Wisconsin, USA, 2020. HCW, healthcare worker; HCW-F, HCW’s family member.

We sequenced viral RNA isolated from nasopharyngeal swab samples from patients 1 and 2, the HCW, and the family member. To determine whether the HCW most likely acquired infection in the healthcare setting or in the community, we compared consensus SARS-CoV-2 sequences from these 4 persons.

All 4 samples were prepared for sequencing by using the ARTIC protocol (https://artic.network/ncov-2019/ncov2019-bioinformatics-sop.html) and were sequenced on an Oxford Nanopore GridION device (Nanopore Technologies, https://nanoporetech.com/products/gridion). Consensus sequences were derived by using a modified version of the ARTIC bioinformatics protocol (https://www.protocols.io/view/ncov-2019-sequencing-protocol-bbmuik6w), which analyzes data after 100,000 reads have been obtained from each sample (analysis pipelines are available at GitHub, https://github.com/katarinabraun/SARS-CoV-2_sequencing/tree/master/Pipelines_to_process_data/Nanopore_pipeline_ARTIC). 

The sequence from the HCW was identical to that of the HCW’s family member but distinct from that of patients 1 and 2 (Figure 2). Although we cannot with absolute certainty exclude the possibility that the HCW was infected by another asymptomatic, untested hospitalized patient, the identical virus sequences from the HCW and the HCW’s family member provide strong circumstantial evidence for a chain of virus transmission outside of the hospital.

**Figure 2:**
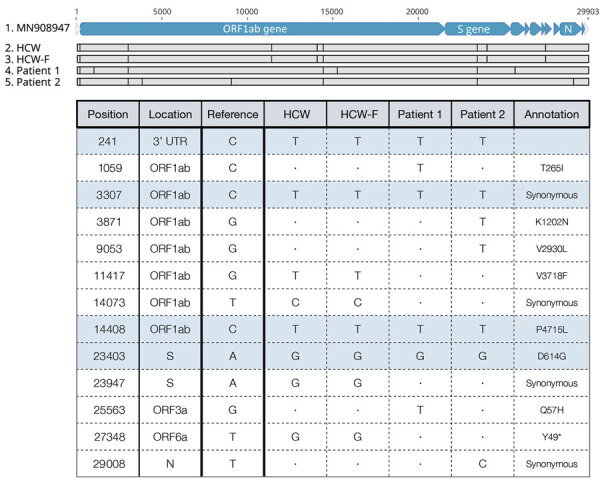
Severe acute respiratory syndrome coronavirus (SARS-CoV-2) consensus-level single-nucleotide variants (SNVs) from investigation of SARS-CoV-2 infection in HCW, Madison, Wisconsin, USA, 2020. The top alignment image depicts the SARS-CoV-2 genome for all persons evaluated in this investigation and highlights SNVs identified relative to the original SARS-CoV-2 reference isolate from Wuhan, China (GenBank accession no. MN908947.3). The table contains additional information about each of these SNVs. Light blue shading indicates A2a clade-defining mutations. Dots indicate identity with reference sequence. Asterisk indicates a tyrosine-to–stop codon change. HCW, healthcare worker; HCW-F, HCW’s family member; ORF, open reading frame; UTR, untranslated region.

Within 2 days of the positive SARS-CoV-2 test result for the HCW, sequencing of the virus identified the probable source of infection as community transmission. This finding offers reassurance to HCWs caring for patients with COVID-19 that appropriate PPE may protect against hospital-acquired SARS-CoV-2 infection. Conversely, had sequencing demonstrated nosocomial transmission, that would have provided an impetus for revisiting infection control strategies. On the basis of these results, sequencing of SARS-CoV-2 from HCWs and known contacts, within and outside of patient care settings, should be an essential component of a comprehensive strategy to protect the health of HCWs and other frontline workers.
